# Characterization of *Tenebrio molitor* Larvae Protein Preparations Obtained by Different Extraction Approaches

**DOI:** 10.3390/foods11233852

**Published:** 2022-11-29

**Authors:** Alkmini-Anna Gkinali, Anthia Matsakidou, Adamantini Paraskevopoulou

**Affiliations:** Laboratory of Food Chemistry and Technology, School of Chemistry, Aristotle University of Thessaloniki, 54124 Thessaloniki, Greece

**Keywords:** *Tenebrio molitor*, protein extraction, physicochemical properties, functional properties, secondary structure

## Abstract

Edible insects have recently attracted research attention due to their nutritional value and low environmental footprint. *Tenebrio molitor* larva was the first insect species to be classified by European Food Safety Authority (EFSA) as safe for human consumption. However, it is thought that the incorporation of edible insect as an ingredient in a food product would be more appealing to consumers than being visible. The aim of the present study was to determine the physicochemical properties of the larvae meal and protein concentrates. Different methods to extract and recover proteins from defatted (DF) *Tenebrio molitor* larvae were applied; i.e., alkaline extraction (DF-ASP); isoelectric precipitation after alkaline extraction (DF-AIP); and NaCl treatment (DF-SSP), and the obtained protein fractions were characterized. The DF-ASP exhibited the highest protein extraction/recovery efficiency (>60%), while it was the most effective in decreasing the interfacial tension at the oil/water (*o/w*) interface. The DF-AIP had the highest protein content (75.1%) and absolute values of ***ζ***-potential and the best ability to retain water (10.54 g/g) and stabilize emulsions at pH 3.0. The DF-SSP protein preparation had the highest oil binding capacity (8.62%) and solubility (~88%) at acidic pHs and the highest emulsifying activity (~86 m^2^/g). Electrophoresis of the protein preparations revealed proteins with different molecular weights, while the protein secondary structure was dominated by *β*-structures and *α*-helix. Protein concentrates with different properties were able to be recovered from *Tenebrio molitor* larvae, that could affect their interactions with other food ingredients and their behavior during processing or storage. These findings would be valuable guidance for the technological exploitation of larvae protein preparations in the development of food formulations.

## 1. Introduction

Western civilization has banned insects from the daily diet, even though there are references to entomophagy (the habit of eating insects) in ancient times, as reported by Aristotle, Pliny the Elder, and other philosophers, and historians, as well as in the literature of many religions [1]. The modern Europeans and North Americans mostly associate insects with food spoilage, unhealthy storage conditions, and primitive behavior [2] in contrast to other ethnicities in Asia, Africa, and Latin America. However, this attitude against entomophagy seems to be changing, especially after the severe impact of climate change on humanity and the environment, which also affects the food supply chain, and, inevitably, food security [1]. People, nowadays, tend to seek natural food with a minor environmental footprint, which also promotes good health [3]. In the context of searching for new food sources that provide sufficient protein and lipid contents, vitamins, minerals, and other micronutrients, edible insects have been proposed to be a viable and sustainable solution. Edible insects are generally considered safe as they are already consumed by millions of people around the globe, and their farming has been proved to consume less energy and water, require limited land, and emit much fewer greenhouse gases compared to conventional livestock farms [1]. European Union (EU) Regulation (EU) 2015/2283 [4] categorizes edible insects as “novel food”. Recently, on 13 January 2021, the European Food Safety Authority (EFSA) published safety approvals for dried *Tenebrio molitor* larva (yellow mealworm) [5]. Moreover, on 25 August 2021, EFSA published a scientific opinion on the “safety of frozen and dried formulations” from the whole *T. molitor* larva [6] as a novel food pursuant to the above Regulation, and EU legislation has already harmonized and authorized the placing of dried *T. molitor* larva on the market as a novel food (Regulation 2021/882) [7]. The *T. molitor* larva is also allowed as fish, poultry, and pig feed according to the Regulation (EU) 2021/1372 [8]. These EU arrangements open the door for wider insect commercialization in the food and feed industry. 

The *T. molitor* larva is one of the most industrialized farming edible insect species and already has large farming facilities in European countries, such as France and Netherlands [9]. Smaller farms are also being created all over Europe, as a response to the emerging sector trend and the opportunities that arise. *T. molitor* farming is rather simple and requires limited equipment and resources [1]. Several studies have demonstrated that *T. molitor* larva is a good source of protein, lipids, carbohydrates, certain vitamins, and minerals [9]. However, despite its nutritional value, the most significant obstacle to incorporating it into the diet of Europeans is overcoming the feeling of disgust against it [2]. Influenced by the outcomes of many surveys that the best way to achieve consumer acceptance is by exposing insects to food products in a non-visible form [10] and in order to increase insect-based food acceptance, the research has pivoted towards the preparation of *T. molitor* larva protein concentrates or meal powders [11,12]. Defatting is considered a significant process step because *T. molitor* larva is rich in fat and thus can interfere with the protein concentrate preparation and create off-flavors due to lipid oxidation [9]. Likewise, many investigators have obtained *T. molitor* larva protein extracts achieving relatively high yields and high protein content [11,13,14,15]. Despite the efforts to increase extraction yield and to obtain extracts with high protein content, there is limited information regarding their physicochemical properties, which are essential for their further exploitation in preparing food products with good and stable sensory and textural properties. 

Based on the above, the objectives of the present study were to obtain different *T. molitor* larvae protein fractions with different properties, by altering the protein extraction or recovery conditions. The extraction yield, protein content, molecular weight, solubility, *ζ*-potential, oil/water surface tension, water and oil binding capacity, and emulsion activity and stability of the protein preparations were measured. Moreover, their protein secondary structure was determined and an effort to associate its different elements with the observed physicochemical properties was also performed. 

## 2. Materials and Methods

### 2.1. Materials

*T. molitor* in the larval stage was purchased from a local breeder (Aeiforia, Koufakis, Chania, Greece). The larvae were thoroughly sieved and washed to dispose of frass and substrate residues. The clean larvae were then freeze-dried (−55 °C, 50 Pa) by using a laboratory-scale freeze dryer (Scanvac CoolSafe Pro SL12a02, Labogene, Allerød, Denmark) and ground into flour by a blender Multiquick System 100 (BRAUN, Frankfurt, Germany). The resulting meal (LM) was stored at −18 °C until further use, to avoid any chemical, physical, and microbial degradation. Sulfuric acid, hydrochloric acid, and boric acid were purchased from ChemLab (Queens, NY, USA). Hexane, methanol, acetic acid, sodium hydroxide, potassium bromide, Folin-Ciocalteu reagent, tartaric acid, Tris-glycine, Tris-hydrochloric acid, sodium hydrogen phosphate, sodium dihydrogen phosphate, sodium dodecyl sulfate (SDS), ammonium persulfate (APS), Coomassie Brilliant Blue R-250, bromophenol blue, hydrochloric acid, sodium hydroxide, and sodium azide were products of Sigma Aldrich (Hamburg, Germany). Glycerol, acrylamide, bis acrylamide, and bromocresol green were supplied by Panreac AppliChem (Darmstadt, Germany). Copper sulfate and sodium carbonate were purchased from Merck (Darmstadt, Germany), potassium sulfate from Honeywell Fluka (Seelze, Germany), and methyl red from May & Baker Ltd. (Dagenham, UK). The electrophoresis protein marker (Blue Star Prestained Protein Marker) was purchased from Nippon Genetics (Düren, Germany). All chemicals were of analytical grade and chemicals/reagents used for electrophoresis were of electrophoretic grade. The corn oil was purchased from the local market. Deionized water was used in all experiments.

### 2.2. Defatting

The removal of larvae fat was performed with hexane by a three-step extraction process. The LM was first mixed with hexane in a mass/volume ratio of 1:5 *w/v*, and the mixture was stirred by using a rotary shaker (KS 4000 ic control, IKA, Königswinter, Germany) for 1 h (150 rpm, 25 °C). The resulting slurry was then centrifuged (Rotina 35, Hettich, Düsseldorf, Germany) at 9000× *g* for 10 min at 25 °C and the supernatant organic phase was decanted. The sediment was re-mixed with hexane and the procedure was repeated twice. The final sediment was left overnight at room temperature to remove residual hexane and the obtained defatted larvae powder (DF) was stored at −18 °C until further use. The fat extraction yield was calculated according to Equation (1) [15]:

(1) $$\;\%\;\mathrm{fat}\;\mathrm{extraction}\;\mathrm{yield}=\frac{m_{e}}{m_{o}}\times 100$$

where *m_e_*: solids of extract (g) and *m_o_*: solids of the initial sample (g).

### 2.3. Protein Extraction and Recovery Process 

**Figure 1:**
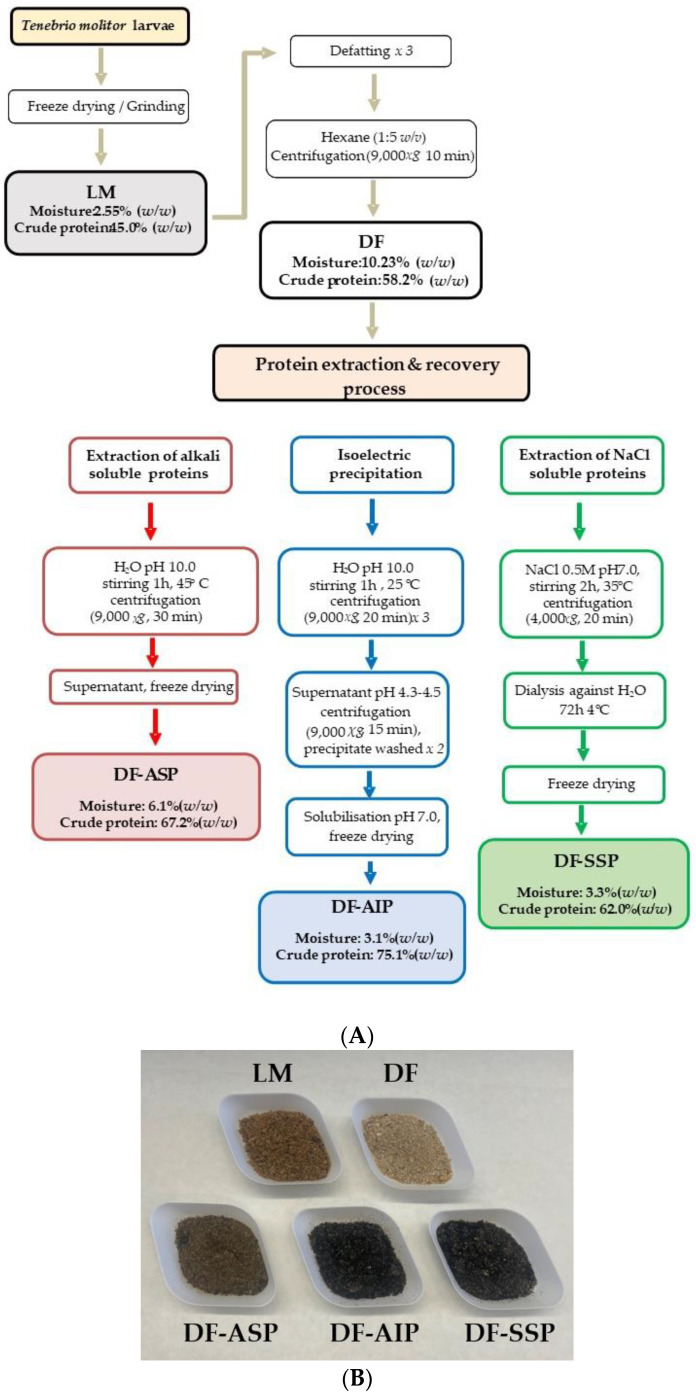
(**A**) Flow diagram of *T. molitor* larvae protein extraction and recovery processes, and (**B**) photographs of the obtained larvae meal powder (LM), defatted powder (DF), and the protein preparations obtained with alkali extraction (DF-ASP), alkali extraction followed by isoelectric precipitation (DF-AIP), and salt assisted extraction (DF-SSP).

Different protein fractions were extracted and recovered by applying three approaches that rely on protein solubility dependence on the change of pH or ionic strength, in accordance with preliminary experiments and literature data, i.e., (1) protein alkaline extraction of DF (DF-ASP), (2) isoelectric precipitation after alkaline extraction of DF (DF-AIP), and (3) NaCl protein extraction of DF (DF-SSP) (Figure 1A). The final protein preparations (Figure 1B), obtained by lyophilization under the same conditions as before, were stored at −18 °C in a desiccator, until further use. 

#### 2.3.1. Extraction of Alkali Soluble Proteins

The protein extraction of soluble proteins under an alkaline environment was performed according to the method described by Azagoh et al. [13], with minor modifications. Briefly, 20 g of the DF was mixed with 300 mL of distilled water (1:15 *w/v*) under magnetic stirring. The pH value of the mixture was immediately adjusted to 10.0 using a 0.1 or 1 M NaOH solution and stirred for 1 h at 45 °C. The pH value was monitored periodically and adjusted to 10.0 if necessary. The supernatant was recovered after centrifugation (9000× *g*, 30 min, 4 °C) and, after pH adjustment to 7.0, using a 0.1 or 1 M HCl solution, was freeze-dried under the same conditions as before to obtain Defatted Alkali Soluble Proteins (DF-ASP). 

#### 2.3.2. Isoelectric Precipitation of Alkali Extracted Proteins 

Isoelectric precipitation was performed after protein alkaline extraction of DF, according to the method described by Santhosh et al. [14] with some modifications. Briefly, 20 g of DF was dispersed in 300 mL distilled water (1:15 *w/v*) and the pH value was adjusted to 10.0, using a 0.1 or 1 M NaOH solution, followed by mechanical stirring for 1 h at room temperature and centrifugation at 9000× *g* (20 min, 4 °C). Three extraction cycles were performed, and the obtained supernatants were combined into one. The pH value was then adjusted to ~4.4 ± 0.1 with 0.1 or 1 M HCl solution. The precipitate was recovered (9000× *g*, 15 min, 4 °C) and washed twice with distilled water. The pH value of the dispersion was adjusted to 7.0, using a 0.1 or 1 M NaOH solution centrifuged (9000× *g*, 10 min, 4 °C) and the sediment was freeze-dried under the same conditions as before to obtain Defatted Alkali extracted Isoelectric Precipitation Proteins (DF-AIP).

#### 2.3.3. Extraction of NaCl Soluble Proteins

Salt soluble proteins were extracted from defatted larvae according to Pissia, Matsakidou, Paraskevopoulou, and Kiosseoglou [16] with some modifications. Briefly, 20 g of DF was dispersed in 200 mL of NaCl solution (0.5 M, pH 7.0) in a 1:15 ratio (*w/v*) and mechanically stirred for 2 h at 35 °C. The suspension was then centrifuged (4000× *g*, 20 min, 4 °C) and the supernatant was dialyzed against water (pH 7.0) at 4 °C for 72 h by employing a dialysis tubing (D-0655) with a diameter of 25 mm and retention capacity of 12,000 Da (Sigma, St. Luis, MO, USA). The protein extract was freeze-dried to obtain Defatted Salt Soluble Proteins (DF-SSP).

### 2.4. Characterization of Raw Materials and Protein Preparations

#### Proximate Composition Analysis of the Raw Material

Moisture, protein, ash, and fat content were determined according to standard AOAC methods (i.e., AOAC 945.32, AOAC 2011.11, AOAC 923.03, and AOAC 920.32, respectively) [17]. Moisture and ash contents were measured gravimetrically. Fat content of LM and DF was determined by using the Soxhlet extraction method. Protein content was determined by Kjeldahl method (conversion factor: 4.76 for whole larvae and 5.60 for protein extracts) [18]. The chitin content of DF was determined according to the two-step method described by González, Garzón, and Rosell [19]. In the first one (i.e., demineralization), 10 g of the DF was mixed with 200 mL of HCl solution 1 M (1:15 *w/v*) at 85–90 °C for 50 min, under mechanical stirring in order to remove catechols, followed by centrifugation (3000× *g*, 5 min) and double washing with deionized water to remove the excess of HCl. In the second step (i.e., deproteinization), the obtained precipitate was suspended in 200 mL of NaOH solution 1 M (1:20 *w*/*v*)) under mechanical stirring at 85–90 °C for 35 min, to remove proteins. The mixture was then filtrated under vacuum using a Buchner funnel (filter paper of 125 mm pore size), washed several times to remove the NaOH excess, and then dried in an oven at 100 °C overnight. The resulting residue was purified chitin, and its content was calculated by weight. Non-protein matter, composed of ash, fat, and soluble constituents such as carbohydrates, was calculated by difference from protein and moisture content. 

Fatty acid methyl esters (FAMEs) were prepared by direct transesterification of the lipids extracted from the sample by applying the Soxhlet extraction method. FAMEs separation was performed in an Agilent 6890A gas chromatograph equipped with a split-splitless injector and a flame ionization detector (FID) according to the conditions described by Zakidou and Paraskevopoulou [20]. The fatty acids were identified by comparing their retention time with those of a standard FAME mixture (Supelco, Bellefonte, PA, USA), and their percentage was calculated based on the total area of the peaks present. The fatty acid profiles of the larvae are presented in Table S1.

Finally, the mineral content of *T. Molitor* was analyzed as reported by Manousi and Zachariadis [21] (Table S2), by employing a Perkin-Elmer Optima 3100XL axial viewing ICP-AES system equipped with a cyclonic spray chamber, and a GemTip cross-flow nebulizer.

### 2.5. Molecular Weight Determination by Polyacrylamide Gel Electrophoresis 

The determination of the molecular weight (MW) distribution of the insect proteins was performed according to the sodium dodecyl sulfate-polyacrylamide gel electrophoresis (SDS-PAGE) method [22] with modifications using 10% (*w*/*v*) polyacrylamide gels containing 0.1% (*w*/*v*) SDS. The samples were treated with a 200 mM Tris Buffer Marker containing 8% (*w/v*) SDS, 0.4% (*w/v*) bromophenol blue, 40% (*v/v*) glycerol, and 10% (*v/v*) *β*-mercaptoethanol and, after heating for 5 min for denaturation of proteins, they were placed in the electrophoresis gel along with the Blue Star Prestained Protein Marker. The gels were then loaded on a Mini-PROTEAN 3 cell (Bio-Rad Laboratories, Hercules, CA, USA), and the electrophoresis was conducted at 100–130 V. The resulting gels were stained with Coomassie brilliant blue R-250. 

### 2.6. Fourier Transform Infrared (FTIR) Spectra Analysis

The IRAffinity-1 spectrometer (Shimadzu Fourier Transform Infrared Spectrophotometer, Shimadzu Crop., Kyoto, Japan) was utilized to obtain FTIR spectra of the LM, DF, and the protein preparations. FTIR spectra were recorded in the range of 700 to 4000 cm^−1^ with 64 scans and instrument resolution 4 cm^−1^ over disks prepared with a mixture of sample powder and KBr and KBr disk as a background spectrum. Spectra were atmosphere corrected using IR solution software (version 1.50, Shimadzu Corporation, USA), smoothed and baseline corrected with the aid of Spectragryph optical spectroscopy software version 1.2.15 (Oberstdorf, Germany).

The secondary structure of the samples was determined by deconvoluting the amide I area at (1600–1700 cm^−1^) by calculating the second derivative (Spectragryph optical spectroscopy software version 1.2.15). The contribution of the amide I constituents that corresponded to different secondary structures obtained by the second derivative, was then calculated by curve fitting of Gaussian curves with the aid of MagicPlot Student 2.9.3 software and expressed as area% of the amide I area. 

The assignment of the secondary structures was according to previous literature data as follows: *β*-antiparallel-sheet with a peak center in the region 1613–1637 cm^−1^, and *β*-parallel-sheet in the region 1682–1695 cm^−1^, a random coil with a peak center in the region 1637–1645 cm^−1^, *α*-helix with a peak center in the region 1645–1662 cm^−1^, *β*-turn with a peak center in the region 1662–1682 cm^−1^ [23,24].

### 2.7. Protein Solubility

The method described by Lowry et al. [25] was performed to determine the protein solubility of the final protein preparations. The protein preparations were dispersed in deionized water (0.1% *w*/*v*) and the pH value was adjusted to 10.0, under magnetic stirring for 1 h. The pH value was then properly adjusted to values from 10.0 to 2.0 under stirring and the mixture was centrifuged (10,000× *g*, 10 min) to recover the supernatant that contained the solubilized protein fraction. The protein content was determined over the absorption at 750 nm (Hitachi U-2000 spectrophotometer, Tokyo, Japan), with bovine serum albumin (BSA) as the standard protein. The solubility was expressed as the percentage of the protein content of the supernatant to the total protein content of the sample.

### 2.8. ζ-Potential

The *ζ*-potential was measured to evaluate the protein surface charge as a function of pH using the Zetasizer Nano system (Malvern Instruments, Malvern, UK). Larvae protein preparations were dispersed in 0.01 M phosphate buffer (pH 7.0, 25 °C) at a protein concentration of 0.1% (*w*/*v*) and the pH value was adjusted from 2.0 to 10.0. The measurements took place at 25 °C. 

### 2.9. Surface Activity 

For the measurement of the ability of the protein preparations to reduce the interfacial tension at oil/water interfaces, the samples were first dispersed in 2 mM phosphate buffer (pH 7.0) at 1% (*w*/*v*) protein concentration under stirring for 1 h at room temperature. The initial dispersions were then diluted covering a range of concentrations from 0.001 to 1% (*w*/*v*). A layer of corn oil (10 mm) was then gently added, and the interfacial tension was measured at 25 °C as a function of time until equilibrium was reached (~24 h). Surface activity was determined using a tensiometer (Kruss, Hamburg, Germany) based on Du Noüy ring method [26]. The surface tension of the buffer at the *o/w* interface was 72 mN/m at 25 °C. All measurements were taken in triplicate. 

### 2.10. Water and Oil Binding Capacity

The water binding (WBC) and oil binding (OBC) capacity of the LM, DF, and protein preparations were estimated according to the procedure described by Bußler et al. [11] with some modifications. Briefly, 0.1 g of sample was weighed into centrifuge tubes and 2 mL of water or oil was added for WBC or OBC determination, respectively. The suspensions were vortexed for 3 min, left to rest for 30 min, and then centrifuged (4000× *g*, 5 min). After decanting the supernatant, the tubes were put upside-down on filter paper for 45 min and the obtained sediment was weighed. *WBC* and *OBC* were calculated according to Equation (2):

(2) $$WBC\;or\;OBC=\frac{\left(w_{2}-w_{1}\right)}{w_{0}}$$

where $w_{2}$ is the mass of the final sediment (after decanting the oil or water), $w_{1}$ is the initial mass of the sample and *w*_0_ is the dry mass of the sample.

### 2.11. Emulsion Activity and Stability

Emulsifying properties were evaluated according to the method of Pearce and Kinsella [27] with some modifications. Briefly, 10 mL of corn oil was added to 30 mL of protein solution (0.1% *w/v*) under mechanical stirring (1 min) (RW14, IKA, Staufen, Germany). The pH values of 30 mL of samples were adjusted to 3.0, 7.0, and 9.0 and the rough emulsions were then homogenized by a mechanical homogenizer (UltraTurrax T25, IKA, Staufen, Germany) for 3 min. An emulsion aliquot of 50 mL was dispersed in 5 mL of 0.1 *w/v* SDS solution after 0 and 10 min of the homogenization. The dispersions were vortexed for 5 s. The absorbances at 500 nm of the diluted emulsions were obtained immediately after the emulsion formation (*A*_0_) and after 10 min (*A*_10_). 

The emulsifying activity index (EAI, m^2^/g) was calculated by Equation (3):

(3) $$\mathrm{EAI}=\frac{2.303\cdot 2\cdot A_{0}\cdot \mathrm{dilution}\;\mathrm{factor}}{C\cdot \varphi \cdot 10,000}$$

where *C* = 0.001 g protein/mL, *φ* is the oil volume fraction (0.25), and dilution factor = 100 (50 mL emulsion diluted with 5 mL 0.1% *w*/*v* mL SDS solution).

The emulsion stability index (ESI, %) was calculated according to Equation (4):

(4) $$\mathrm{EAI}(\min)=\frac{A_{0}}{A_{0}-A_{10}}\cdot 10$$

where ${\mathrm{EAI}}_{10}$ is the emulsifying activity at 10 min and EAI_0_ is the emulsifying activity at 0 min.

### 2.12. Statistical Analysis

All measurements were repeated in triplicate. The results were analyzed by one-way analysis of variance with Duncan’s test, which was performed by IBM SPSS Statistics 27.0.1 (Armonk, NY, USA). The significance of difference among the mean values was indicated at the 95% confidence level.

## 3. Results and Discussion

### 3.1. Chemical Composition

The chemical composition of major components of the *T. molitor* larvae samples is shown in Table 1. Fat removal, as expected, resulted in an increased (*p* < 0.05) protein (58.2% *w*/*w* d.b.) and ash (5.76% *w*/*w* d.b.) content in DF (45.0, and 4.69% *w/w* d.b., respectively). The chitin content of DF was found 11.07 ± 1.26% *w/w* (d.b.) higher compared to the novel food that was subjected for evaluation by EFSA (~6% *w*/*w* w.b.) [5]. The protein content of LM (45% *w*/*w* d.b.) is consistent with previous data (44.8% *w*/*w* d.b.) reported by Janssen et al. [18] who investigated and proposed a corrected Nitrogen-to-Protein factor that was also adopted in the present study. The corrected factor excludes nitrogen derived from other molecules, such as chitin, which are present in relatively high amounts in *T. molitor* larvae. Older publications reported protein contents in the range of 41–66% *w/w* (d.b.) for *T. molitor* larvae [9,11,13,15,19], and up to 77.4% *w*/*w* (d.b.) [15] for the defatted *T. molitor* larvae powder. However, the protein content in all these studies was probably overestimated, as a Nitrogen-to-Protein factor of 6.25 was applied. Thus, the present study demonstrated similar protein contents compared to the above-mentioned studies if the protein content was calculated with the same Nitrogen-to-Protein factor. Moreover, the fat content of LM was about 23.07 ± 0.15% *w/w* (d.b.), while the fat extraction and recovery process resulted in reducing the fat content of DF down to 1.97 ± 0.08% *w/w* (d.b.) (yield of ~91 ± 2%) comparable to the results reported by Bußler et al. [11] (19.1 and 2.8% *w/w* d.b. fat content of *T. molitor* larvae powder and defatted sample, respectively). As found, the fatty acids profile of LM (Table S1) was dominated by oleic (18:1) (44.82%) followed by linoleic (18:2n−6) (26.35%) and palmitic (16:0) (21.14%) acid, similarly to other reports [9]. Variations in FA contents between available literature data may be attributed to different rearing conditions [9]. The content of ash in LM was 4.69 ± 0.05% *w/w* (d.b.) and was comparable to previously reported data (4.25–4.90% *w/w*) [15,19]. The values of heavy metals of LM (Table S2) appeared to be below the maximum permitted levels (0.050 and 0.10 mg/Kg wet weight) for meat products (for cadmium and lead, respectively) that were dictated by the Commission Regulation (EC) 1881/2006 [28], as no maximum levels of heavy metals are set for insects as food. LΜ was rich in magnesium (239.26 mg/100 g), while the levels of iron and zinc were considerably higher (12.70 and 23.99 mg/100 g, respectively) than those reported in the literature [9], which may be due to potential differences in breeding conditions.

Not surprisingly, the protein extracts were more concentrated (*p* < 0.05) in protein compared to DF. The protein content of the preparations varied significantly (*p* < 0.05). DF-AIP exhibited the highest protein content (75.1% d.b.) followed by DF-ASP (67.2% d.b.) and DF-SSP (62.0% d.b.). The efficiency of the protein extraction process was higher (62.5%) in the case of DF-ASP, while the recovery of the protein by the precipitation method resulted in lower (*p* < 0.05) efficiency (32.0%). It seems that the application of isoelectric precipitation increases the recovery of the proteins after alkali extraction from the larvae. The efficiency of the extraction of DF-SSP was 12.5%, significantly lower than the DF-AIP extraction yield. The above yields are comparable to those reported in the literature for alkali extraction from untreated larvae (60%) [13] or followed by isoelectric precipitation (22%) [11]. The lower yield of DF-SSP extraction may be attributed to the lower content of larvae in salt soluble proteins compared to the total protein fraction. However, even though the extraction and recovery yield of DF-SSP is low, the resulting crude protein content of DF-SSP is almost as high as the DF-ASP.

### 3.2. SDS-PAGΕ Electrophoresis

**Figure 2:**
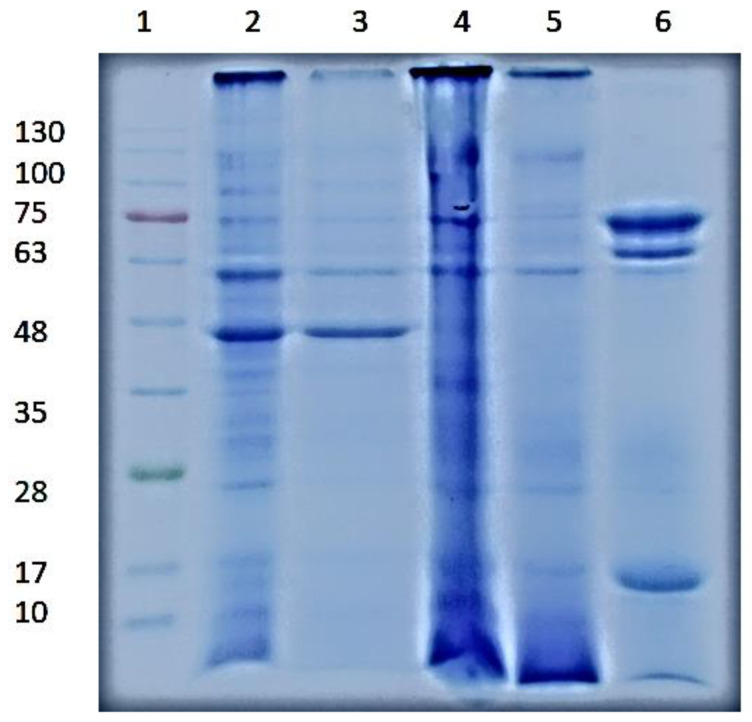
The linearly enhanced (sharpness) electropherogram image of the molecular weight distribution of *T. molitor* fractions determined by SDS–PAGE (samples from left to right: Lane 1: molecular marker, Lane 2: LM, Lane 3: DF, Lane 4: DF-AIP, Lane 5: DF-ASP, and Lane 6: DF-SSP). Original image is presented in Figure S1.

SDS-PAGE electropherograms of LM, DF, DF-AIP, DF-ASP, and DF-SSP are shown in Figure 2. Proteins with molecular weights between 10 and 130 kDa were obtained. The mass of protein in each sample pocket was approximately the same (150 μg/mL), meaning that the total mass of protein obtained in each lane was the same. Three major groups of protein bands corresponding to MWs of <17 kDa, 17–48 kDa, and 48–130 kDa were distinguished. According to Yi et al. [12], the bands ranging from 8.5 to 13 kDa could probably emanate from anti-freeze type of proteins, including hemolymph proteins with a MW of ~12 kDa. The bands in ~28 kDa could possibly originate from T. *molitor* cuticle proteins, e.g., chymotrypsin-like proteinase (24 kDa) [12]. Finally, the bands from 48 to 130 kDa might be ascribed to enzymes and other proteins, e.g., melanization inhibition protein (43 kDa), *β*-glycosidase (59 kDa), trypsin-like proteinases (59 kDa) as well as to other proteins involved in the synthesis of melanin (85 kDa) [12]. The LM and DF (lines 2 and 3, respectively) showed bands corresponding to the range of the MWs mentioned above, with those in the case of DF being weaker. The DF-AIP and DF-ASP samples did not exhibit differences in their quality features, as they both yielded bands at 10, 17, 28, and >62 kDa. Τhe DF-SSP exhibited some faint bands also at around 28–35 kDa, likely linked to the salt-soluble tropomyosin (34 kDa), and some stronger ones at the range of 62–75 kDa. According to Kim et al. [29], the water-soluble proteins of *T. molitor* larvae have MWs generally below 25 kDa and usually between 10 and 15 kDa. Yi et al. [12] reached a similar conclusion that the water-soluble proteins had MWs < 14 kDa. 

### 3.3. FT-IR Spectroscopy

**Figure 3:**
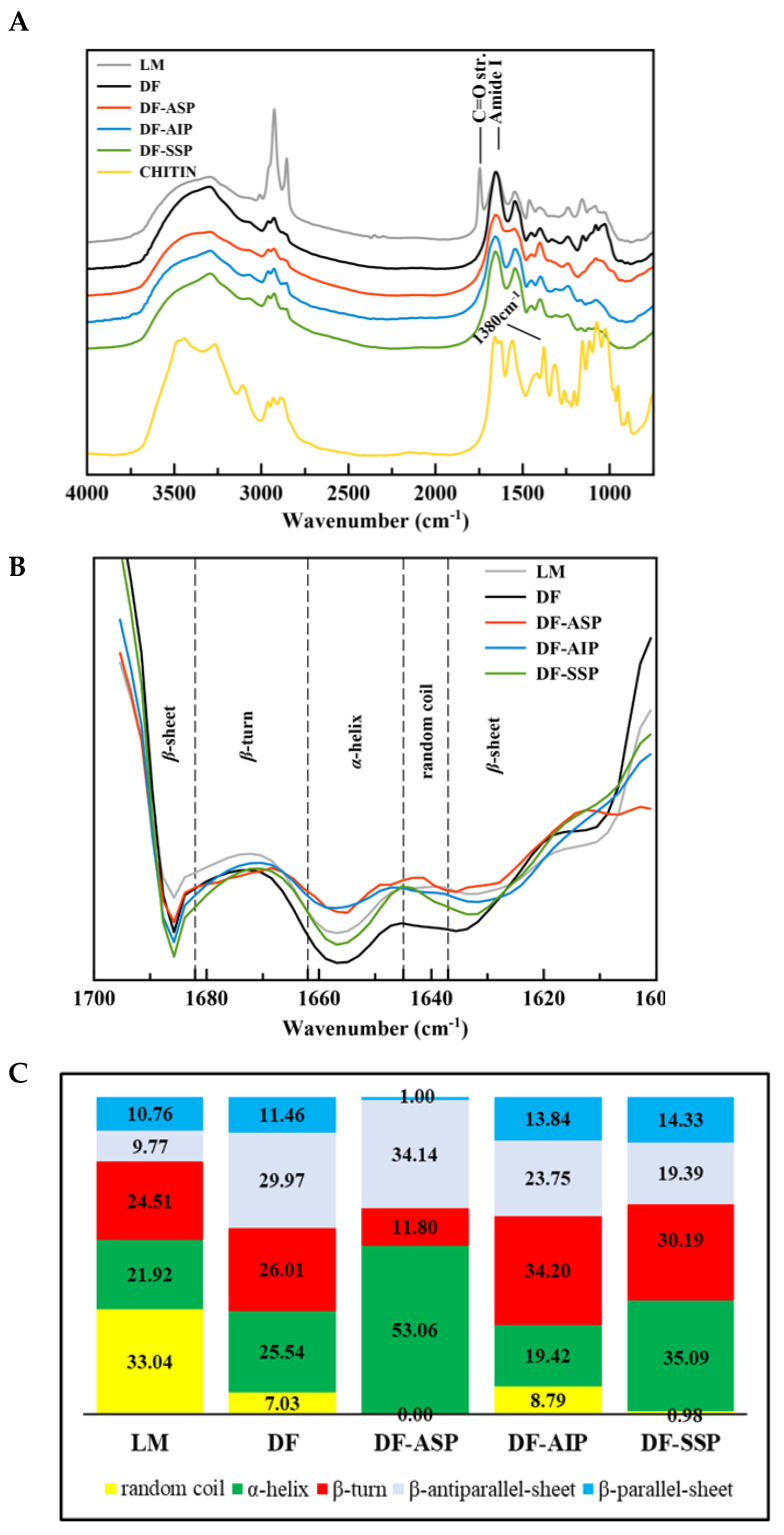
(**A**) Normalized FTIR spectra; (**B**) second derivative of the Amide I region; (**C**) contribution (%) of the different conformations to the protein secondary structure of the protein preparations. Notations as in Table 1.

As expected, based on their chemical composition, the FT-IR spectra of the LM, DF, DF-ASP, DF-AIP, and DF-SSP were similar (Figure 3A). The region 4000–2500 cm^−1^ in all spectra was dominated by a wide peak (Amide A) ranging from 3600 cm^−1^ to 3000 cm^−1^, attributed to intermolecular H-bonded N-H and O-H stretching vibration, characteristic for hydrophilic materials [23]. The bands at 1655 and 1546 cm^−1^ are characteristic of proteins and are ascribed to Amide I (80% C = O stretch, 10% C-N stretch) as well as to Amide II (60% N-H bend, 30% C-N stretch, and 10% C-C stretch) [23]. However, the pure chitin (Ch) isolated from the LM also yielded absorption peaks at 1650, 1620, and 1550 cm^−1^ (Figure 3A), in correspondence with the literature data [30]. The peak at 1240 cm^−1^ can be assigned to Amide III and N-H bending [31]. The spectrum of LM yielded unique peaks that were attributed to the presence of fat, i.e., the peak at 3008 cm^−1^, corresponding to the C-H stretching vibration of the *cis*-double bond (=CH), is associated with the unsaturated fatty acids, the intense peaks at 2925 and 2854 cm^−1^ correspond to the symmetric and asymmetric vibrations of the aliphatic -CH_2_ bonds of lipids, and the peak at 1745 cm^−1^ corresponds to the carbonyl group of the esters of lipid triacylglycerols [32]. 

The peak at around 1380 cm^−1^ (Figure 3A), evident in the spectrum of *T. molitor* pure chitin (Ch), was only found in the spectrum of LM and was attributed to C–H vibrations [33]. The spectrum of Ch presented high-intensity peaks in the region 860–1200 cm^−1^. These peaks were also evident in the spectra of all samples, being more intense in the case of LM and DF (Figure 3A), as a result of the protein applied extraction and recovery processes. The high-intensity peak at 1080 cm^−1^ found at the Ch spectrum, also appeared in the spectra of all samples being more distinct in the case of LM and DF, has been previously connected to the asymmetric stretching of the C-O-C structure in chitosan samples [33].

#### Protein Secondary Structure

Protein solubility [34], digestibility [35], and techno-functional properties [36] have been related to the secondary conformation of the food proteins and its determination may provide helpful insight regarding possible protein applications. Τhe second derivative of the Amide I region (1600–1700 cm^−1^) was calculated (Figure 3B) to deconvolute to the specific protein secondary structure elements of the samples. It is evident from Figure 3B that defatting, protein extraction, and recovery processes resulted in changes in the protein conformation, elucidated by differences in their intensities, yield of new peaks, and/or shifts of the peak centers, which is probably due to the reordering and partial unfolding of protein chains as a response to either defatting or alkali extraction, isoelectric precipitation, and salt presence. The ratios (%) of the protein conformation structures contribution were calculated by the areas of the Gaussian curves fitted to the Amide I region (Figure S2) and are presented in (Figure 3C). LM protein secondary structure exhibited higher content in random coil (33.04%) followed by *β*-turn (24.51%), *α*-helix (21.92%), and combined *β*-sheet structures (20.56%) (Figure 3C). Defatting favored the formation of *β*-sheet structures from 9.77 up to 29.97% and 10.76 up to 11.46%, corresponding to the *β*-antiparallel-sheet and *β*-sheet, in the expense of the random coil content which was decreased down to 7.03%. The *α*-helix and *β*-turn structures content were also increased by 3.5% and by 1.5%, respectively, after defatting. In general, the combined *β*-sheet structures content was higher in all defatted samples compared to the LM. According to Carbonaro et al. [35], *β*-sheet structures have been correlated with limited digestibility of plant and animal protein sources.

Alkali extraction had a drastic effect on the secondary structure of the protein. The proteins of the DF-ASP were characterized by highly ordered structure, dominated by *α*-helix (53.06%), while the contribution of *β*-turn structures dropped down to 11.80% and random coil was not detected. The combined *β*-sheet structures also decreased from 41.43 (DF) down to 35.14% (DF-ASP), mainly composed of *β*-antiparallel sheet structures (34.14%) rather than *β*-parallel-sheet structures (1.00%). 

The application of isoelectric precipitation to the DF-ASP led to the recovery of a protein fraction that was dominated by *β*-structures (37.59% of the combined *β*-sheet conformations and 34.20% of the *β*-turn structure), while the random coil was the least favored structure (8.79%). Previous literature data regarding alkaline extraction followed by isoelectric precipitation of *T. molitor* proteins [37] reported remarkably higher content in unordered structures (47.5%), similar content in *β*-sheet structures (38.6%), 13.9% content in *β*-turn structures and total absence of *α*-helix conformation. The differences in the secondary structure of the protein recovered by isoelectric precipitation may be attributed to the lower temperature of the alkali extraction process applied in the present study (i.e., 25 °C) compared to the process reported by Jiang et al. [37] (i.e., 80 °C). The DF-SSP sample favored almost equally the formation of *α*-helix (35.09%), *β*-turn (30.19%) and total *β*-sheet content (33.72%) conformations. The random coil structure was nearly detectable (0.98%). 

### 3.4. Protein Solubility at Different pH Values

**Figure 4:**
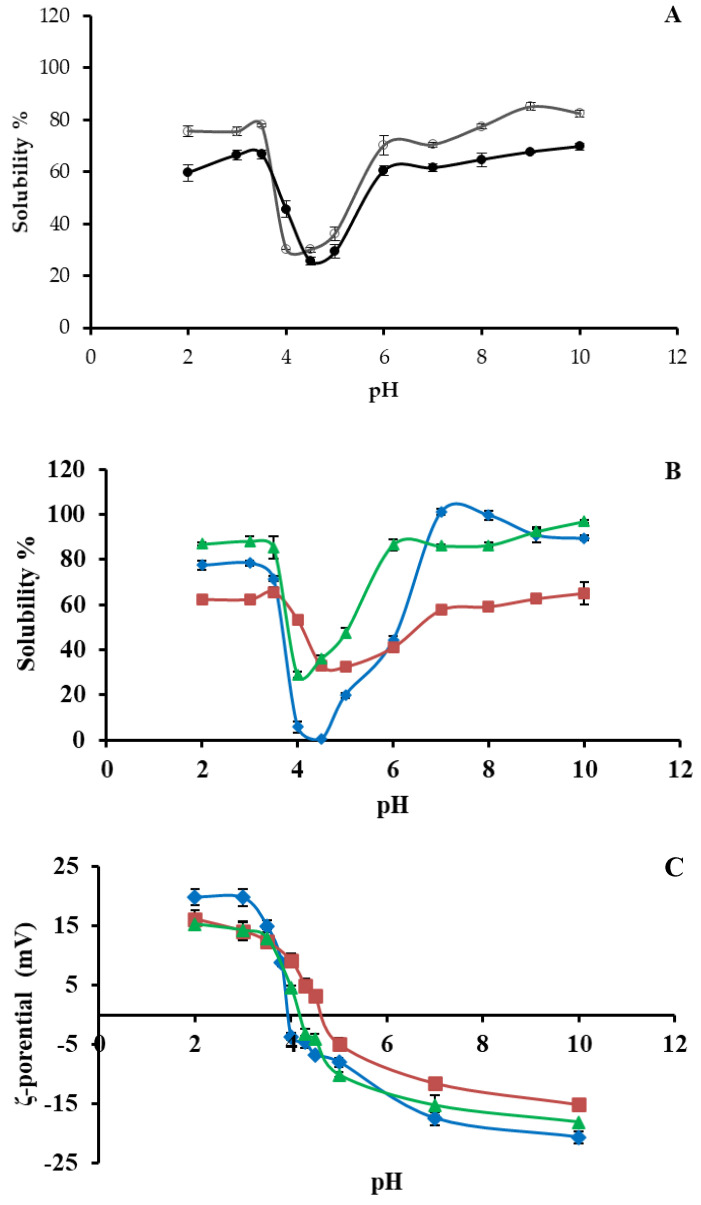
Effect of pH value on (**A**) protein solubility of LM (**o**), and DF (●), (**B**) protein solubility, and (**C**) *ζ*-potential of DF-ASP (■), DF-AIP (◆), and DF-SSP (▲). Notations as Table 1.

Solubility is an important property of proteins that can affect their functionality and, therefore, is a key factor for designing protein extraction processes and new protein-based products [13]. The protein solubility of the samples as a function of pH is presented in Figure 4A. The curves of all samples exhibited the characteristic U shape depending on the pH values. LM and DF exhibited a similar solubility trend throughout the pH values range, with that of the DF remaining lower compared to LM (*p* < 0.05). Available data regarding the effect of defatting on the protein solubility of *T. molitor* larvae are contradictory between published studies. Borremans, Bußler, Sagu, Rawel, and Schlüter [38] reported that defatting decreased the solubility of larvae meal, which is consistent with the findings of the present study. On the other hand, Bußler et al. [11] observed opposite trend, i.e., meal flour exhibited remarkably lower solubility values compared to the defatted flour. The solubility of the LM and DF increased in both acidic and alkaline regions, reaching the maximum value of 85.10% at pH 9.0 for LM and 69.75% at pH 10.0 for DF proteins. The minimum solubility (30.36 and 25.80%) was noticed at pH values of 4.0 and 4.5 for LM and DF, respectively. Borremans et al. [38] reported solubility (~52–55%) of *T. molitor* meal and defatted mealworm powder at pH 10.0 and minimum solubility (3%) at pH 4.0. Bußler et al. [11] reported that ~70% of the *T. molitor* flour and defatted flour protein was soluble at pH 10.0 and only ~5.0% and ~20.0% were soluble at pH 4.0, respectively. 

As shown in Figure 4Β, all samples demonstrated high solubility values in both the acidic and alkaline pH regions. DF-AIP was fully soluble (~100%) at the pH value range of 7.0–8.0. A fraction of 65.06% and 97.05% of DF-ASP and DF-SSP proteins, respectively, were soluble at a pH value of 10.0. The solubility values of the protein preparations in the alkaline region increased as the content in *α*-helix conformation decreased (Figure 3A), which is in line with the findings of Tan et al. [34]. In the acidic environment (2.0–3.5), DF-SSP protein solubility was remarkably high (up to ~88%). The isoelectric precipitation resulted in a ~78% solubility, while the DF-ASP’s soluble fraction reached 65.03%. At pH values between 4.0 and 6.5, DF-AIP presented ~30% lower solubility compared to the DF-ASP and DF-SSP. This difference could be attributed to the presence of insoluble, high MW proteins (Figure 2) and, possibly, to the presence of insoluble aggregates formed during the extensive structure modification and denaturation of the molecules during protein recovery by isoelectric precipitation. Moreover, the presence of less-hydrophobic water-soluble aggregates may, partly, justify the higher solubility of DF-ASP and DF-SSP proteins in the isoelectric region compared to the proteins present in the DF-AIP. Furthermore, Azagoh et al. [13] reported high surface hydrophobicity of the *T. molitor* larvae soluble proteins which may result in the formation of protein structures with “hidden” hydrophobic zones, thus resulting in the formation of soluble aggregates with low surface hydrophobicity. The lower solubility of DF-AIP was evidently due to the recovery of the insoluble proteins at the narrow pH range between 4.3 and 4.5. Other researchers have previously stated that the maximum solubility of *T. molitor* larvae powders ranges between 74 and 100% at pH values between 8.0 and 9.0 [11,15]. 

The isoelectric point (*p*I) of all samples was determined in the pH range between 4.0 and 5.0, where the minimum values of solubility were detected (30.36% at pH 4.5, 25.80% at pH 4.5, 32.47% at pH 5, 0.30% at pH 4.5, and 28.89% at pH 4.0 corresponding to LM, DF, DF-ASP, DF-AIP, and DF-SSP). Zhao et al. [15] and Bußler et al. [11] also reported that proteins extracted from freeze-dried *T. molitor* larvae exhibited *p*I within the pH range of 4.0 and 5.0 owing to considerably fewer repulsions between the protein molecules, leading to their aggregation [13]. A wider range of *p*I (3.0 to 5.0) was noted by Azagoh et al. [13] suggesting that thermal treatment of the larvae might have affected the protein solubility. 

### 3.5. ζ-Potential at Different pH Values

The measurement of *ζ*-potential aims to estimate the accumulated charge on the surface of the protein molecules. The electrostatic interactions between charged particles provide valuable information relating to protein adsorption phenomena. As shown in Figure 4C, the *ζ*-potential curve was similar for all three protein preparations. As expected, the *ζ*-potential decreased as the pH value increased, demonstrating positive values at acidic and negative values at alkaline pH regions. This trend of the curve could be attributed to protonation and deprotonation of the amino acids of the protein, at acidic and alkaline pH values, respectively [13].

At pH value 2.0, the *ζ*-potential of the DF-ASP and DF-SSP proteins was ~15 mV (*p* > 0.05) while the *ζ*-potential of the DF-AIP was significantly higher (19.88 mV, *p* < 0.05). At pH 7.0, by increasing order of absolute values, the *ζ*-potential of DF-ASP, DF-SSP, and DF-AIP was −11.49 mV, −15.19 mV, and −17.4 mV, respectively (*p* < 0.05). The same trend was observed at pH value 10.0, where DF-ASP, DF-SSP, and DF-AIP showed *ζ*-potential values −15.1, −18.02, and −20.61 mV, respectively (*p* < 0.05). 

Generally, the *ζ*-potential results are in accordance with the solubility findings, as at high absolute *ζ*-potential values (either positive or negative) the charged chains tend to repel each other and not form aggregates. As shown in Figure 4C, the *ζ*-potential approached zero value at pH values 4.6, 3.9, and 4.2 for the DF-ASP, DF-AIP, and DF-SSP, respectively, in line with the respective low solubility values. The higher negative charge of DF-AIP in the alkaline region in comparison to the DF-ASP and DF-SSP led to higher electrostatic repulsions between the protein molecules and can justify the increased solubility of DF-AIP proteins at high pH values (Figure 4B). Azagoh et al. [13] observed a similar trend for soluble protein concentrates from *T. molitor* larvae, i.e., at pH 2.0 the concentrates showed *ζ*-potential about ~30 mV, at pH 10.0 *ζ*-potential ranged from −20 to −35 mV, while it approached zero at pH between 3.0 and 3.5. Likewise, Jiang et al. [37] reported *ζ*-potential equal to −14.40 mV (at pH 7.0), for protein obtained with alkali extraction combined with isoelectric precipitation, close enough to the value obtained in the present study (−17.4) (Figure 4C).

### 3.6. Surface Activity at Oil/Water Interfaces

Surface activity is an important physical property that affects protein functionality. The presence of surface-active molecules, such as proteins, at the air/water or oil/water interface, is related to their ability to reduce surface tension contributing to better foaming or emulsifying ability. 

**Figure 5:**
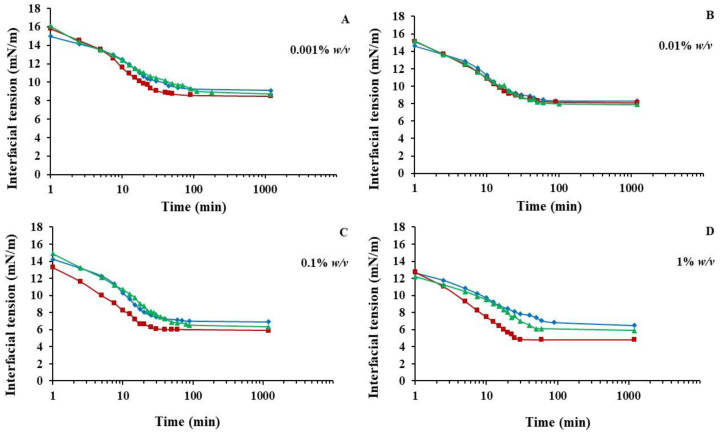
(**A**–**D**) Effect of adsorption time on the interfacial tension of DF-ASP (■) DF-AIP (**♦**), and DF-SSP (▲) at different protein concentrations. Different letters indicate significant (*p* < 0.05) differences between means. Notations as in Table 1.

The surface tension change at the oil/water interface with time for DF-ASP, DF-AIP, and DF-SSP samples at different protein concentrations is shown in Figure 5. Generally, the interfacial tension of the protein solutions at equilibrium decreased with increasing protein concentration from 0.001 to 1% *w/v* (*p* < 0.05), while the rate of interfacial tension development increased [39]. As can be seen, the interfacial tension values did not differ significantly between the samples (*p* > 0.05) at low protein concentration levels but only at the higher ones. Specifically, at 0.001 (Figure 5A) and 0.01% *w/v* (Figure 5B), the protein preparations exhibited a similar rate of interfacial tension decrease through time. However, DF-ASP was more effective in decreasing the interfacial tension when protein was added at higher levels, i.e., 0.1 (Figure 5C), and 1% *w/v* (Figure 5D), compared to the DF-AIP and DF-SSP, resulting in the equilibrium state of ~4.85 mN/m after 30 min. On the contrary, the equilibrium state (6.10 mN/m) was reached after 50 min for DF-SSP and after 90 min for DF-AIP (6.85 mN/m), which exhibited a greater value of interfacial rate decrease. Low MW proteins, present in the DF-ASP, are more likely to rapidly diffuse to the oil/water interface reducing the interfacial tension values compared to the larger MW of the DF-SSP and even more compared to the proteins present in the DF-AIP (Figure 2). It has been previously reported for chickpea protein isolates that isoelectric precipitation may alter the conformation of the proteins towards a more unstructured order [26] which may be the cause of the weakened surface activity of DF-AIP compared to the DF-ASP, despite the fact that DF-ASP solubility at pH value 7.0 is much less than that of DF-AIP. Moreover, the effectiveness of DF-ASP in rapidly decreasing the interfacial tension could be attributed to its highly ordered structure (*α*-helix) (Figure 3C), which is associated with the reduction of the time needed for the reordering of protein molecules at the interface and the establishment of interactions with the oil phase [40]. In general, low content in random conformation is considered to lead to faster adsorption of the protein molecules [40].

It is worth noting that the interfacial tension values of all samples remained below 9 mN/m lower, compared with those of whey protein isolate (~13 mN/m), sodium caseinate (11.5 mN/m), and lentil protein isolate (12.5 mN/m LPI) solutions (1% *w/v*) [41]. This observation may be an indication of better emulsification activity of *T. molitor* protein preparations than that of conventional protein sources and their potential utilization for the formation and stabilization of emulsion systems.

### 3.7. Water/oil Binding and Emulsifying Capacity

The effect of applying different protein extraction/recovery methods from the *T. molitor* larvae on protein MW, secondary structure, and physicochemical properties of the obtained preparations is expected to influence their techno-functional properties and future exploitation in food technology. WBC and OBC refer to the ability of proteins to retain water or oil, respectively, and are related to features, such as texture, tenderness, and juiciness and may be affected by processing [42]. The WBC and OBC (Table 2) of the LM were found to be 3.06 and 3.0 g/g, respectively. Defatting resulted in reducing WBC down to 2.39 g/g (*p* < 0.05). On the contrary, OBC remained practically unchanged (3.44 g/g, *p* > 0.05) after fat extraction. The ability of LM and DF to retain water or oil was similar to that reported for flours coming either from *T. molitor* or other insect species (1.29–2.82 g/g) [9,43] as well as other protein sources (i.e., freeze-dried beans: WBC 2.00–3.55 g/g, OBC 4.88–7.71 g/g) [44].

Concerning the protein preparations, WBC was remarkably high in the case of DF-AIP (10.54 g/g, *p* < 0.05) and comparable to those reported for snail meat concentrate (10.0 g/g) [16] and soya protein isolate 12.40 g/g [45]. The high absolute charge of the DF-AIP at pH > 6.0 (Figure 4C) as well as structure modification because of the applied extraction process may have induced the exposure of water binding sites of the protein chains favoring protein–water interactions [46]. The DF-ASP exhibited zero WBC since this protein fraction was fully soluble in water. All protein preparations exhibited higher WBC values than those reported by Zielińska et al. [43] (1.29 g/g) and Bußler et al. [11] (0.8 g/g dry mass). The OBC values of the DF-ASP, DF-AIP, and DF-SSP were significantly higher than LM and DF, reaching up to 5.79, 4.87, and 8.62 g/g, respectively, and higher than those reported by Zielińska et al. [43] (1.71 g/g). Differences may be attributed to the different extraction and recovery methods or experimental conditions.

The indices of emulsifying activity (EAI) and emulsion stability (ESI) measure the capacity of a protein to form and stabilize an emulsion system. EAI determines the amount of oil that can be emulsified by a unit mass of protein and ESI indicates the ability of the formed emulsion to resist change during the defined period [47]. Table 2 shows the effect of pH value on the EA and ESI of the DF-ASP, DF-AIP, and DF-SSP. At pH 3.0 (time = 0), DF-SSP had a far superior ΕA (85.67 m^2^/g), which was ~2 times higher than DF-AIP (45.75 m^2^/g) and ~3 times higher than DF-ASP (27.82 m^2^/g). The higher solubility of DF-SSP at pH 3.0 compared to DF-ASP and DF-AIP (Figure 4B) may have enhanced its emulsifying properties [48]. The higher value of pH (7.0) gave rise to the EAI values of the DF-ASP, DF-AIP, and DF-SSP up to 147.82, 112.7302, and 145.37 m^2^/g, respectively, in compliance with their interfacial behavior (Figure 5), even though the solubility values at pH 3.0 and 7.0 were similar for DF-ASP and DF-SSP (Figure 4B). The changes obtained in the secondary structure during extraction and recovery processes (Figure 3C) may have resulted in the uncovering of hydrophobic domains which enhanced the EAI values. Moreover, the ESI of DF-SSP at pH 3.0 was found to be significantly lower (18.98%, *p*<0.05) than DF-AIP and DF-ASP (37.58 and 26.96%, respectively). On the contrary, at pH 7.0, the ESI did not significantly vary among the samples and was lower than the values reported by Zielińska et al. [43] for the protein preparation from the edible insect *Gryllodes sigilattus* (38.3%). 

## 4. Conclusions

The study demonstrated that the technο-functional properties of protein preparations obtained by *T. molitor* larvae differed depending on the extraction approach. According to electrophoresis and FTIR results, different extraction procedures resulted in protein preparations with different molecular weights and secondary structures. All the samples showed high solubility in the acidic and alkaline pH region. The protein extraction procedures enhanced the WBC and OBC values, except for DF-ASP, which is soluble in water. All samples presented better surface activity in comparison to conventional proteins. In terms of emulsifying properties, at pH value 3.0 the DF-AIP showed the highest ESI, while at pH value 7.0 the three protein preparations presented similar ESI values. Given that *T. molitor* protein preparations exhibited different properties, they could be exploited in various applications. Denaturation kinetics data would also be useful to understand, and manipulate accordingly, the factors affecting the properties of the protein preparations to achieve the development of acceptable products based on these powders. Furthermore, the stability of the powders against processing, such as heating, and storage parameters are currently being under investigation to establish data regarding the production and market line. In general, for future work, it would be worth investigating aspects ensuing from the coexistence of protein preparations and other food components for developing insect protein-fortified food formulas. 

## Figures and Tables

**Table 1:** Proximate chemical composition ^1^ of LM, DF, and protein preparations.

Sample	Moisture (% *w/w*)	Protein Content ^2^ (% *w/w* d.b.)	Non-Protein Matter Content ^3^ (% *w/w* d.b.)
LM	2.55 ± 0.08 ^a^	45.0 ± 0.64 ^a^	42.45
DF	10.23 ± 0.24 ^d^	58.2 ± 1.19 ^d^	47.97
DF-ASP	6.10 ± 0.14 ^c^	67.2 ± 0.72 ^c^ (62.5 ± 4.23) ^c^	61.10
DF-AΙP	3.08 ± 0.03 ^b^	75.1 ± 0.16 ^e^ (32 ± 0.36) ^b^	72.02
DF-SSP	3.27 ± 0.29 ^b^	62.0 ± 0.80 ^b^ (12.5 ± 2.98) ^a^	58.73

^1^ Values are mean ± standard deviation (*n* = 3); values in the same column with the same superscript letter do not differ significantly (*p* > 0.05). ^2^ Values correspond to extraction and recovery (in parentheses) yields. Notations as in Figure 1: LM, larvae meal; DF, defatted; DF-ASP, defatted-alkali extracted proteins; DF-AIP; defatted-alkali extracted/isoelectric precipitation protein; DF-SSP, defatted-salt soluble protein. ^3^ Calculated by difference from protein and moisture content.

**Table 2:** Water/oil binding capacity and emulsifying activity and stability of the protein preparations.

Sample			pH 3.0			pH 7.0		
	WBC (g/g)	OBC (g/g)	EAI_0_ m^2^/g	EAI_10_ m^2^/g	ESI %	EAI_0_ m^2^/g	EAI_10_ m^2^/g	ESI %
LM	3.06 ^c^	3.00 ^a^						
DF	2.39 ^b^	3.44 ^b^						
DF-ASP	0.00 ^a^	5.79 ^d^	27.82 ± 2.70 ^a^	17.47 ± 3.24 ^a^	26.96	147.82 ± 6.03 ^b^	33.84 ± 0.21 ^a^	14.58
DF-AΙP	10.54 ^e^	4.87 ^c^	45.75 ± 2.82 ^b^	33.59 ± 2.99 ^b^	37.58	112.02 ± 3.55 ^a^	35.19 ± 2.23 ^a^	12.99
DF-SSP	3.54 ^d^	8.62 ^e^	85.67 ± 5.45 ^c^	40.44 ± 2.09 ^c^	18.98	145.37 ± 1.57 ^b^	42.31 ± 2.56 ^b^	14.11

Values are mean ± standard deviation (*n* = 3); values in the same column with the same superscript letter do not differ significantly (*p* > 0.05). Notations as in Table 1.

## Data Availability

The data presented in this study are available on request from the corresponding author.

## Supplementary Materials

The following supporting information can be downloaded at: https://www.mdpi.com/article/10.3390/foods11233852/s1, Figure S1: The molecular weight distribution of *T. molitor* fractions determined by SDS–PAGE (original version). Notations as in Figure 2; Figure S2: Curve fitting of the Amide I region FTIR spectrum for calculating the secondary structure contribution to the protein secondary conformation of A. LM, B. DF, C. DF-ASP, D. DF-AIP, E. DF-SSP. Notations as in Table 1; Table S1: Fatty acid profile of *Tenebrio molitor* larvae meal (LM); Table S2: Mineral content of *Tenebrio molitor* larva meal (LM) (mg/100 g).
