# Supplementary material for: Characterization of Tenebrio molitor Larvae Protein Preparations Obtained by Different Extraction Approaches
Source: Foods. 2022 Nov 29;11(23):3852. doi: 10.3390/foods11233852 (PMC9737764; doi:10.3390/foods11233852)
Supplement: Supplementary file 1 [file foods-11-03852-s001.zip › foods-2029886-supplementary1.pdf]

Characterization of *Tenebrio molitor* larvae protein preparations obtained by different extraction approaches

Alkmini-Anna Gkinali [1], Anthia Matsakidou [1], Adamantini Paraskevopoulou [1,*]

[1] *Laboratory of Food Chemistry and Technology, School of Chemistry, Aristotle University of Thessaloniki, 54 124 Thessaloniki, Greece*

* Correspondence: adparask@chem.auth.gr

Supplemmentary Material

Table S1. Fatty acid profile of *Tenebrio molitor* larvae meal (LM).

**Fatty acids**	**%***
14:0	2.61±0.116
16:0	21.14±0.449
16:1	0.43±0.011
17:0	-
18:0	3.62±0.067
18:1	44.82±0.341
18:2 n-6	26.35±0.175
18:3 n-3	1.03±0.007
20:0	-
20:1 n-9	-
20:3 n-6	-
20:4	-
21:0	-
22:0	-
22:2	-
23:0	-
24:0	-
Total SFA**	27.37
Total MUFA**	4.24
Total PUFA**	27.38
Total omega-3 FA	1.03
Total omega-6 FA	26.35
Ratio omega-6/omega-3	25.55

* Values are given as mean ± SD from triplicate determination. ** SFA: saturated fatty acids (C6:0, C8:0, C12:0, C13:0, C14:0, C15:0, C16:0, C18:0, C20:0, C23:0), MUFA: mono unsaturated fatty acids (C16:1, C17:1, C18:1, C20:1), PUFA: polyunsaturated fatty acids (C18:2, C18:3, C20:5, C22:2), omega 3: omega 3 fatty acids (C18:3, C20:5) και omega 6: omega 6 fatty acids (C18:2).

Table S2. Mineral content of *Tenebrio molitor* larva meal (LM) (mg/100g).

**Minerals**	**mg/100g** *
Cd	<0.02
Cr	0.59±0.170
Cu	0.87±0.227
Fe	12.70±2.754
Pb	<0.02
Mn	0.88±0.082
Zn	23.99±3.096
Al	2.08±0.206
Mg	239.26±27.98
Ca	48.11±7.156

*Values were given as mean ± SD from triplicate determination.

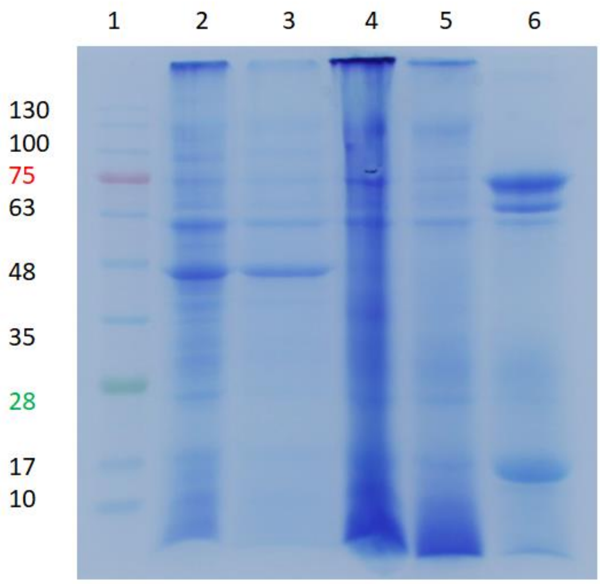

Figure S1. The molecular weight distribution of *T. molitor* fractions determined by SDS–PAGE (original version). Notations as in Figure 2.

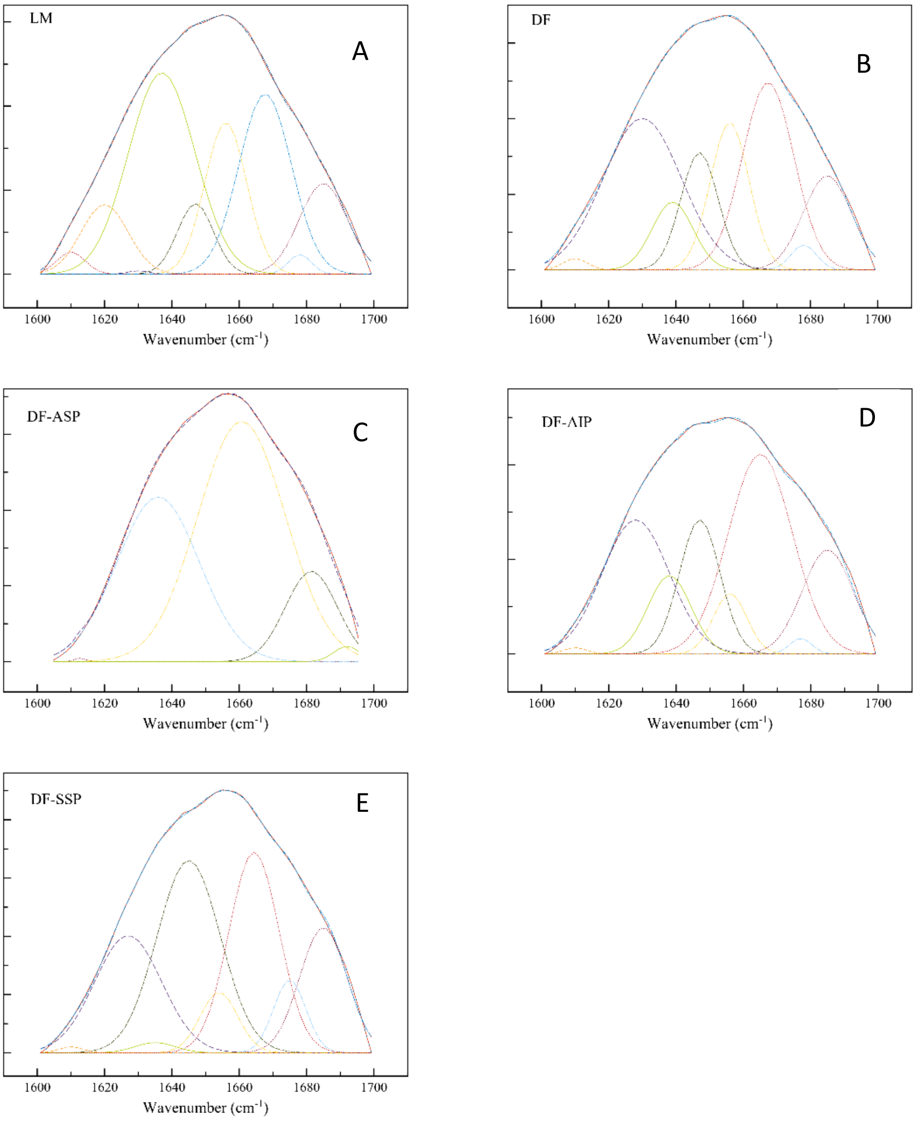

Figure S2. Curve fitting of the Amide I region FTIR spectrum for calculating the secondary structure contribution to the protein secondary conformation of **A.** LM, **B.** DF, **C.** DF-ASP, **D.** DF-AIP, **E.** DF-SSP. Notations as in Table 1.
